# Fabrication of Optical Switching Patterns with Structural Colored Microfibers

**DOI:** 10.1186/s11671-018-2614-2

**Published:** 2018-07-09

**Authors:** Geon Hwee Kim, Taechang An, Geunbae Lim

**Affiliations:** 1 Department of Mechanical Engineering, Pohang University of Science and Technology (POSTECH), Pohang, 790-784 Republic of Korea; 2 Department of Mechanical Design Engineering, Andong National University, Kyungbuk, 760-749 Republic of Korea

**Keywords:** Electrospinning, Hydrothermal growth, Nanostructure, Structural color, ZnO

## Abstract

Structural color was generated using electrospinning and hydrothermal growth of zinc oxide (ZnO). An aligned seed layer was prepared by electrospinning, and the hydrothermal growth time control was adjusted to generate various structural colors. The structural color changed according to the angle of the incident light. When the light was parallel to the direction of the aligned nanofibers, no pattern was observed. This pattern is referred to as an “optical switching pattern.” Replication using polydimethylsiloxane (PDMS) also enabled the generation of structural colors; this is an attractive approach for mass production. Additionally, the process is quite tunable because additional syntheses and etching can be performed after the patterns have been fabricated.

## Background

Structural color has many advantages over pigment (chemical) color. For example, it may be eco-friendly and does not suffer from photochemical degradation. Also, because the color changes according to the observing angle, it is possible to produce various patterns that cannot be produced with conventional pigment colors. These attributes have rendered structural colors of great interest to the textiles, paints, cosmetics, security, and sensors [1–7]. A variety of coloring principles explain the expression of structural color, and recent studies have shown that zinc oxide (ZnO) nanostructures express color by quasi-ordered scattering [8].

Quasi-ordered scattering is determined by the size and spacing of the nanostructures and is colored when the size of the nanostructure is similar and the spacing is constant. Although the diffuse reflectance is presumed to be the main coloring principle of quasi-ordered scattering, the principle of precise coloring has not yet been clarified, and blue, green, and purple are mainly observed [8].

A seed layer is required to fabricate ZnO nanostructures. Hydrothermal growth occurs in the region where the seed layer forms, which is also where structural color is expressed [9–14]. Hydrothermal growth refers to the synthesis of nanostructures in water at 40–80 °C. Therefore, the shape of the pattern is defined by the region of the seed layer. To fabricate optical switching patterns, a nanofiber seed layer is required that is aligned in one direction. To accomplish this, we used electrospinning, which is the most commonly used method for fabricating nanofibers [15–18]. However, collected electrospun nanofibers are usually randomly aligned. Research has been conducted to align nanofibers to minimize the net torque of electrostatic forces applied to the fiber ends [19]. In this way, the nanofibers can be aligned in a floating state (the nanofibers are aligned in the air between the electrodes), and an aligned seed layer can be fabricated by transferring the fabricated nanofibers to the target substrate. In order to produce the wire pattern of microscale without using electrospinning, a complicated patterning process using photoresist must be performed, which is a process that is not only difficult to realize mass production and large-scale as well as increase the process cost.

The fabricated seed layer was made from nanofibers having specific dimensions obtained through hydrothermal growth after heat treatment. ZnO is a highly suitable material for fabricating patterns because of its high refractive index (*n* = 2.0034) and ease of synthesis in various forms. The method of fabrication of structural color patterns using aligned ZnO nanofibers proposed in this study can be applied to create visual patterns, or in sensors for detecting various gases [20–22].

## Experimental Methods

### Materials

Polyvinylpyrrolidone (PVP; AR grade, M.W. 1,300,000) powder was purchased from Alfa Aesar. Ammonia solution (AR grade, 28.0–30.0% (mol/mol)), zinc chloride (AR grade), and zinc nitrate hexahydrate (AR grade) were purchased from Junsei Chemical Co., Ltd. Hydrochloric acid (AR grade) and *N*,*N*-dimethylformamide (DMF; AR grade) were purchased from Sigma–Aldrich. All reagents were used as-received and without further purification.

### Electrospinning Conditions

Electrospinning was performed at room temperature and low humidity (relative humidity, 15–20%). A solution in DMF of 500 mM Zn(NO_3_)_2_ and 0.2 g/mL of PVP (final concentrations) was prepared. The gap between the tip and collector was fixed at 50 mm, and the applied voltage was 6.5 kV. To obtain aligned microwires, parallel aluminum electrodes were fabricated with dimensions of 3 cm in width and 2 cm in height. The nanofibers collected in parallel by an electric field were transferred to a target substrate (glass or silicon wafer).

### ZnO Nanostructure Fabrication

To fabricate a ZnO nanostructure that exhibits structural color, a ZnO seed layer must be prepared by heat treatment (500 °C) of the nanofibers prepared in the previous step. Hydrothermal growth was then used to fabricate nanostructures on the seed layer. To fabricate the ZnO nanostructures, ZnCl_2_ was dissolved in deionized water (DI) at a concentration of 10 mM and maintained at 40–80 °C to initiate the reaction. Ammonia (NH_4_OH) was added to this aqueous solution at a rate of 5 μL/mL, generating OH^−^ and raising the pH of the solution. In this environment, the Zn^2+^ ions quickly precipitated out of solution, which led to the nucleation and growth of ZnO nanostructures. To induce nanostructure synthesis at a constant rate, the reaction was carried out at pH > 10, and the pH of the solution decreased due to a dehydration reaction. Hydrothermal growth can be achieved by further growth of the nanostructures after patterning.

### Patterning of ZnO Microwires

The growth of the nanostructures can be adjusted by using lithography to alter the time during which the seed layer is exposed to the reaction solution. In this study, lithography was performed with the help of masking tape. The masking tape was patterned using a paper cutter (Silhouette Cameo) to cut it into the desired shapes.

### Characterization

The morphology of the ZnO nanostructures was observed by scanning electron microscopy (SEM) using a TESCAN LYRA 3 XMH instrument. Microwires were studied using an optical microscope (model D800; Nikon) equipped with a digital camera (model LV-150; Nikon). A white LED was used as the light source.

### Replication of Pattern Using PDMS

The final fabricated ZnO nanostructure is used as a master mold for replication. Replication is carried out using polydimethylsiloxane (PDMS), which is characterized by being inexpensive, flexible, and optically transparent. First, pre-polymer base is mixed with curing agent 10: 1 and bubbles are removed in a vacuum chamber for 1 h to remove bubbles. Pour over the master mold and cure for 1 h at 65 °C in the oven to complete the replication process.

## Results and Discussion

**Fig. 1:**
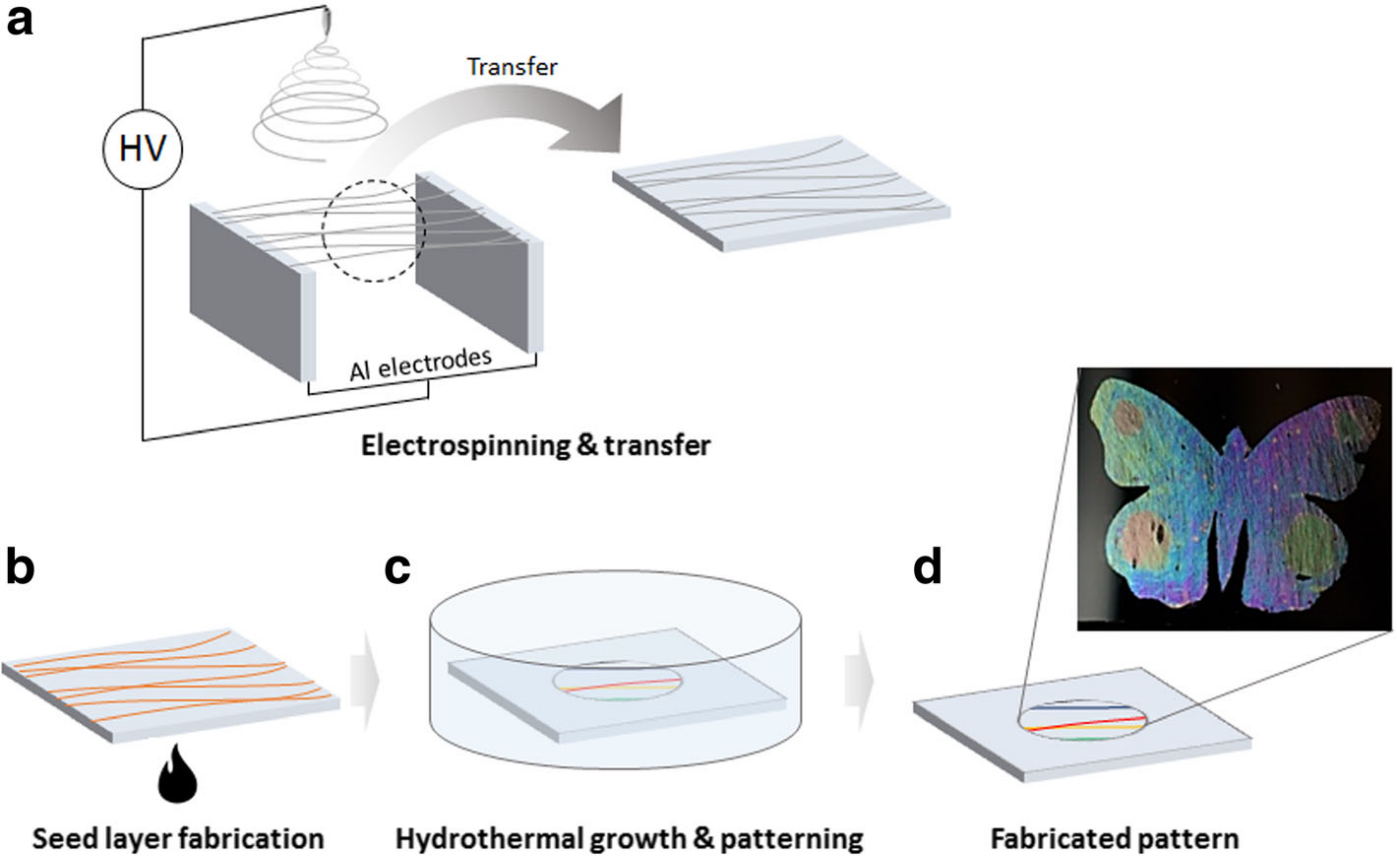
Schematic illustration of the aligned zinc oxide (ZnO) structural color fabrication process. **a** The electrospun nanofiber is collected in a vertical direction between parallel electrodes and transferred to the target substrate. **b** To remove the polymer component of the transferred nanofiber, heat treatment is performed at 500 °C to form a seed layer. **c** Patterning is performed using masking tape, and hydrothermal growth is performed in a constant temperature bath. **d** Removing the masking tape completes the final pattern. (Additional masking and hydrothermal growth allow complex patterns to be created)

Aligned nanofibers are required to produce an optical switching pattern. Nanofibers floating in air are aligned using the parallel collector described above and then transferred to the target substrate (Fig. 1a). The aligned nanofibers on the target substrate are then heat-treated using hot plate (500 °C) to decompose the polymer component and form a thin ZnO seed layer (Fig. 1b). This layer can be grown hydrothermally to obtain the desired structural colors, and the part where the hydrothermal growth occurs can be controlled by patterning the reaction area using a masking technique (Fig. 1c). Then, final pattern is obtained by removing masking tape or additional patterning can be conducted by additional patterning and hydrothermal growth.

**Fig. 2:**
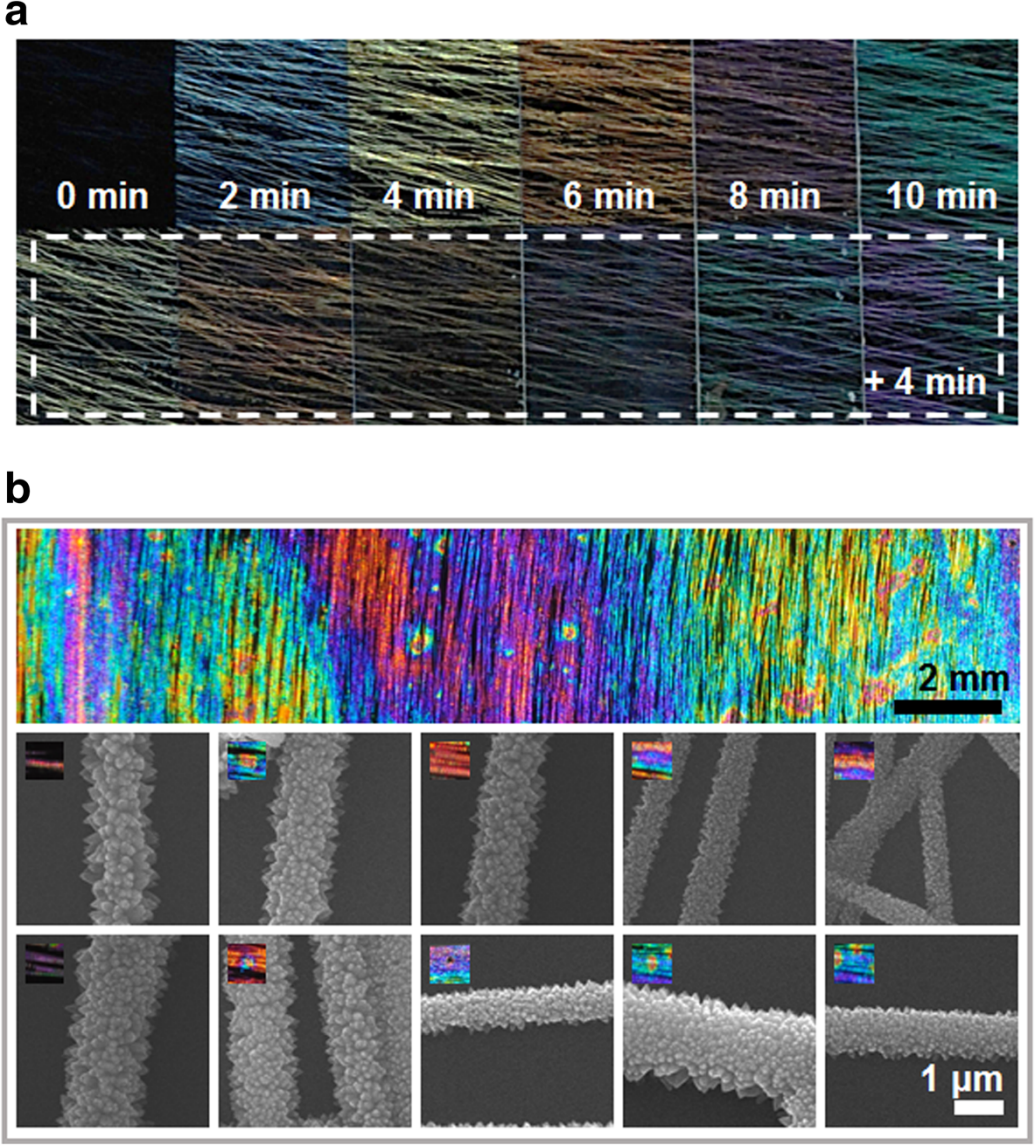
**a** Change of structural color as a function of synthesis time. **b** Optical and scanning electron microscopy images of the nanofibers showing the beautiful structural color pattern attainable with nanofibers fabricated after randomized synthesis times

Figure 2 shows the structural color obtained by varying the hydrothermal growth time of the microwires. As the hydrothermal growth time increases, the thickness of the microwire increases, which causes the optical properties to change. Figure 2a shows the hydrothermal growth time increasing from left to right by 2 min, and the bottom image shows a sample grown for four additional minutes. The structural colored pattern was reproducible for a given synthesis time, and the reaction region was localized using the masking method. Figure 2b shows a sample made to fabricate the sample with randomly bright structural colors. To generate the random colors, a sample with a seed layer was submerged randomly to the hydrothermal growth solution by shaking the sample or spraying the hydrothermal growth solution on the substrate. A random color sample resulted, free of a masking line. The lower SEM image demonstrates that microwires of various dimensions were produced with various colored segments.

**Fig. 3:**
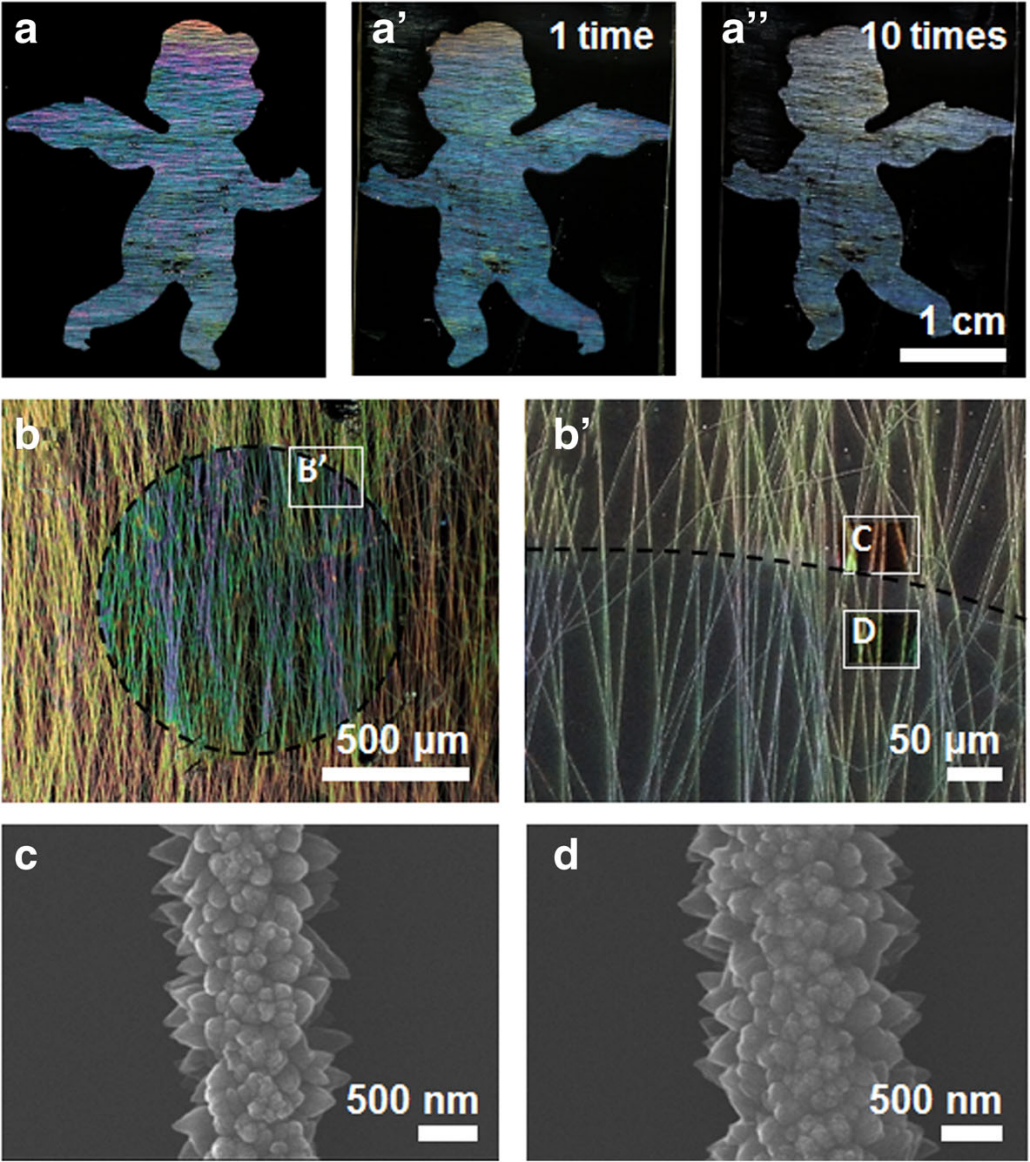
**a** Structural color pattern of an angel and the pattern duplicated 1 time (A**’**) and 10 times (A**”**) using polydimethylsiloxane. **b** Pattern for which two colors were obtained by varying the synthesis time and (**b’**) an image of the edge portion observed with an optical microscope. **c**, **d** Scanning electron microscope images of nanofibers in the outer and inner parts of **b’**

Figure 3 shows how the techniques based on this ZnO microwire fabrication method can be extended. The process of making a structural color using ZnO microwires is not disadvantageous to mass production. The simplest way to mass-produce is to use molds. Figures 3A and A’ show patterns produced using ZnO nanostructured patterns on a glass substrate and duplicated patterns using polydimethylsiloxane (PDMS), respectively. In the replicated pattern using PDMS, the shape of ZnO nanostructure is replicated intact in PDMS (ZnO nanostructure remains on the original glass substrate and does not transferred to PDMS pattern). Figure 3A is a pattern made on glass, while Fig. 3A’ is one made with PDMS; both were fabricated on a transparent substrate. Also, Fig. 3A is an optical image of a sample that has undergone replication 10 times. This confirms that the pattern is well fabricated during the repetitive replication process. In that way, we could observe the structural color when the light coming from the back penetrated the pattern. Since light must pass through the pattern, the transparent substrate must be illuminated from the back, but the light source, the pattern, and the detector to observe do not have to be in a line. The structural color observed in the duplicated sample was similar. Figure 3B shows a sample that demonstrated structural color change through additional growth by restricting the portion to be grown after constructing the structural color. The colors are clearly different from each other. Figure 3B’ shows the result of close examination of the part labeled B′ in Fig. 3B with an optical microscope. Most of the nanofibers are well-aligned in the vertical direction. Clear boundaries are visible between the yellow-colored outer part of the circle indicated by C and the green-colored inner part of the circle indicated by D. Figure 3C, D shows SEM images of C and D, respectively. Further synthesis led to an increase in the overall microwire dimension, but the change in the size of each nanostructure constituting the microwire caused the change in structural color. The SEM image shows that the size of each nanostructure has also been increased, which causes the quasi-ordered scattering.

**Fig. 4:**
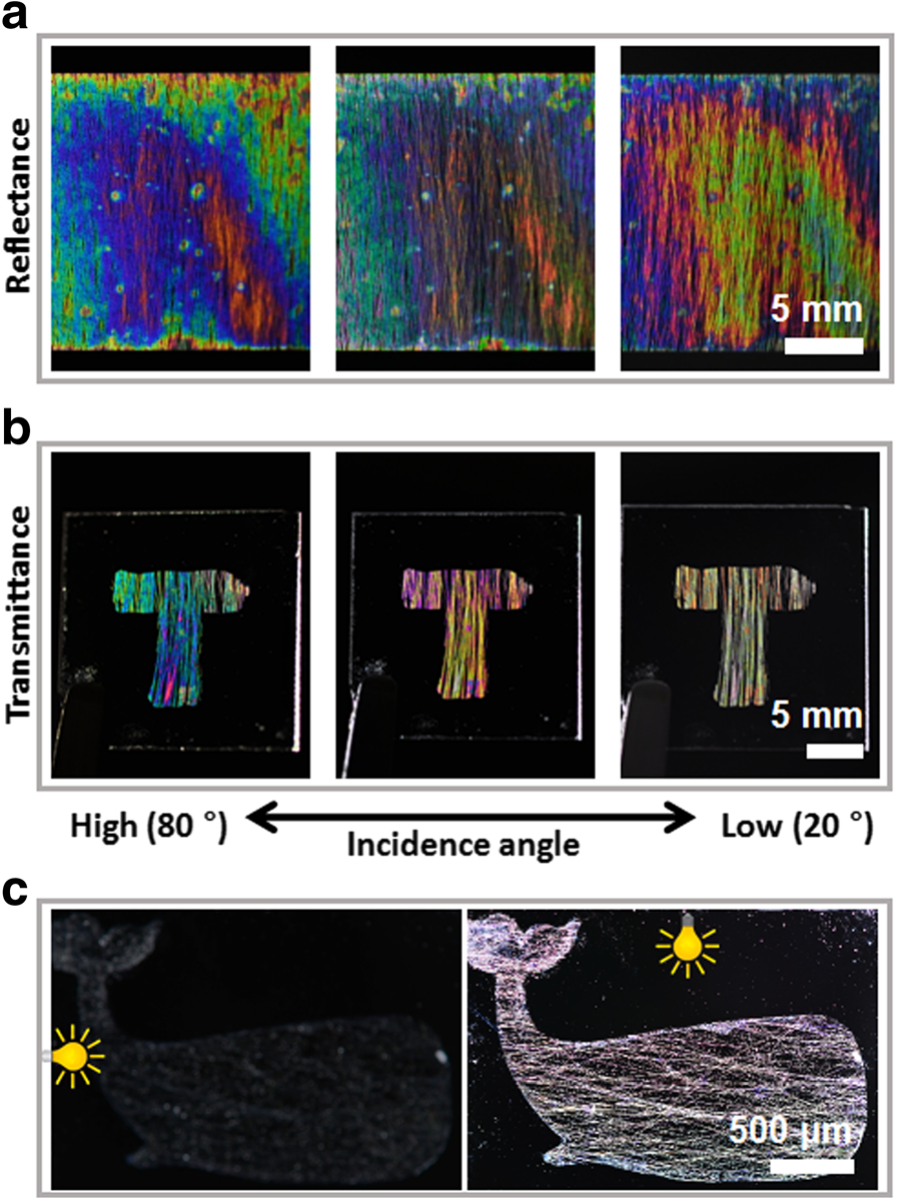
Change of color of a structural pattern as a function of incidence angle on a **a** reflecting substrate and **b** transparent substrate. **c** Effect on pattern visibility by the orientation of incident light relative to the alignment direction of the nanofibers. Left: perpendicular, right: parallel orientation

Structural color changes with viewing angle. Our structures displayed this feature. As noted above, the visible color of a transparent substrate differs from that of a reflecting substrate. With a transparent substrate, light is observed through the substrate, while with a reflecting substrate, light is reflected by the substrate and observed directly by our eyes. In both environments, the characteristic of changing color depending on the angle of observation was retained. Figure 4a shows structural color fabricated on a reflecting substrate (silicon wafer), and Fig. 4b shows structural color made on a transparent substrate (glass). It is evident that the structural color changed according to the angle of incidence. Moreover, not only did the color change with observing angle, but the alignment of the nanofibers enabled the pattern to be made brighter or invisible simply by changing the angle of incidence. If light is incident parallel to the alignment direction of the nanofibers, they hardly reflect the light. On the other hand, if light is incident perpendicularly, it is reflected in many directions, which makes the fiber array easy to see (Fig. 4c). Specifically, light incident in the perpendicular direction is incident on the entire cylindrical portion of the fiber surface, which results in clear visibility because it is reflected in a very wide direction. On the other hand, light incident in the parallel direction can only reflect in a limited direction, so that the total amount of light emitted is inevitably small, making it invisible.

## Conclusion

We fabricated an optical switching pattern using ordered structural coloring nanostructures. The fabricated nanostructures are colored according to the principle of quasi-ordered scattering. Controlling the reaction time affects the size of the nanostructures and thereby the observable colors. We also used electrospinning, which is the most common method for fabricating nanofibers, to form an aligned seed layer to fabricate the alignment pattern. Our fabrication process is highly flexible, because the electrospinning process controlling the position and size of the pattern and the hydrothermal growth controlling the size of ZnO nanostructure can be modified independently. After the process is completed, the pattern can be modified by additional synthesis or etching, and the completed pattern can be mass-produced through replication using PDMS. Large color-changing patterned areas can be produced, for which the color changes according to the viewing direction and the light transmission direction. We successfully fabricated an optical switching pattern, for which the pattern was seen only on one side by aligning the nanofibers along one direction. We expect that our pattern-making method will find widespread applications in applications such as gas sensors and anti-tampering tags.

## Abbreviations

DI: Deionized water
PDMS: Polydimethylsiloxane
PVP: Polyvinylpyrrolidone
SEM: Scanning electron microscopy
ZnO: Zinc oxide
